# Transformation of common wheat (*Triticum aestivum* L.) with *avenin*-*like b* gene improves flour mixing properties

**DOI:** 10.1007/s11032-013-9913-1

**Published:** 2013-08-02

**Authors:** Fengyun Ma, Miao Li, Lingling Yu, Yin Li, Yunyi Liu, Tingting Li, Wei Liu, Hongwen Wang, Qian Zheng, Kexiu Li, Junli Chang, Guangxiao Yang, Yuesheng Wang, Guangyuan He

**Affiliations:** 1 The Genetic Engineering International Cooperation Base of Chinese Ministry of Science and Technology, Chinese National Center of Plant Gene Research (Wuhan) HUST Part, College of Life Science and Technology, Huazhong University of Science and Technology (HUST), Wuhan, 430074 China; 2 The Key Laboratory of Molecular Biophysics of Chinese Ministry of Education, College of Life Science and Technology, Huazhong University of Science and Technology (HUST), Wuhan, 430074 China

**Keywords:** Transgenic wheat, Avenin-like b protein, Mixing properties, Glutenin polymers

## Abstract

Avenin-like b proteins may contribute to the viscoelastic properties of wheat dough via inter-chain disulphide bonds, due to their rich cysteine residues. In order to clarify the effect of the avenin-like b proteins on the functional properties of wheat flour, the functional and biochemical properties of wheat flour were analyzed in three transgenic wheat lines overexpressing the *avenin*-*like b* gene using the sodium dodecyl sulfate sedimentation (SDSS) test, Mixograph and size exclusion-high performance liquid chromatography (SE-HPLC) analysis. The results of the SDSS test and Mixograph analysis demonstrated that the overexpression of avenin-like b proteins in transgenic lines led to significantly increased SDSS volume and improved flour mixing properties. The results of SE-HPLC analysis of the gluten proteins in wheat flour demonstrated that the improvement in transgenic line flour properties was associated with the increased proportion of large polymeric proteins due to the incorporation of overexpressed avenin-like b proteins into the glutenin polymers. These results could help to understand the influence and mechanism of avenin-like b proteins on the functional properties of wheat flour.

**Electronic supplementary material:**

The online version of this article (doi:10.1007/s11032-013-9913-1) contains supplementary material, which is available to authorized users.

## Introduction

Wheat (*Triticum aestivum* L.) is the dominant crop in temperate countries, being used for human food and livestock feed. Wheat flour forms the basis of a variety of food products, largely because of the unique viscoelastic properties of wheat dough conferred by the major storage gluten proteins in the seed endosperm (Shewry 1995, 2009; Shewry et al. 1997). Gluten proteins are classified into gliadins and glutenins. Gliadins are classified as α-, β-, γ- and ω-gliadins on the basis of their electrophoretic mobility in A-PAGE (pH 3.1). Gliadins are monomeric proteins having intra-chain disulphide bonds, with the exception of ω-gliadins, which have no cysteine in their structure (Müller and Wieser 1995, 1997). Glutenins are further divided into high-molecular-weight glutenin subunits (HMW-GS) and low-molecular-weight glutenin subunits (LMW-GS) on the basis of their mobilities on sulfate-polyacrylamide gel electrophoresis (SDS-PAGE) (Shewry et al. 2003). The numbers and distributions of the cysteine residues in glutenin subunits available for inter-chain disulphide bonds are critical in determining the rheological properties of wheat dough (Shewry and Halford 2002; Shewry et al. 1997).

It has been known that the viscoelastic properties of wheat dough are primarily determined by the glutenin fraction of the gluten proteins (Huebner and Wall 1976; Shewry 2009), which comprises HMW and LMW glutenin subunits, linked by inter-chain disulphide bonds to form polymers with a broad molecular-weight distribution. However, the existence of a further class of storage proteins, which were named avenin-like proteins by Kan et al. (2006), may contribute to the functional properties of wheat flour. Kan et al. (2006) characterized two classes of cDNAs encoding avenin-like a and b proteins, based on their nearest relatives identified in databases. The distinguishing feature of avenin-like proteins is that they contain more cysteine residues with the potential to form intra-chain and inter-chain disulphide bonds.

The molecular mass of avenin-like a proteins is about 18 kDa; each avenin-like a protein contains 14 cysteine residues (Kan et al. 2006). Because of the high homology sequence similar to a previously reported “low-molecular weight gliadin” monomer, it may be assumed that cysteine residues of avenin-like a protein mediate seven intra-chain disulfide bonds (Anderson et al. 2001; Clarke et al. 2003; Salcedo et al. 1979). In contrast, avenin-like b proteins contain 19 (typ-b1 and -b2) or 18 (typ-b3) cysteine residues, and do not correspond to any known protein sequences. They differ from avenin-like a proteins in that the mature avenin-like b proteins contain a duplicated sequence of about 120 residues (Kan et al. 2006). The first 18 amino acid residues of each avenin-like b protein have been proved to be a signal peptide while the mature proteins have an average molecular mass of 30 kDa (De Caro et al. 2010). A few years ago, avenin-like b proteins were detected in the glutenin fraction of durum wheat cultivar Svevo (Mamone et al. 2009). The identification of avenin-like b proteins was supported by acquisition of the sequence with a reasonable number of tryptic peptides and the matches between measured and expected molecular weight and pI (Mamone et al. 2009). The detection of avenin-like b proteins in the glutenin fraction together with their high contents of cysteine residues suggested that this type of protein could be integrated into the glutenin polymers by inter-chain disulphide bonds, and possibly contributes to the functional properties of wheat flour.

To support such a hypothesis, Chen et al. (2010) incorporated 10 or 15 mg of the heterologously expressed avenin-like b proteins (containing 19 cysteine residues) into 2 g of flour to evaluate the effect on dough functional properties by the 2 g Mixograph test. They confirmed that incorporation of the heterologously expressed avenin-like b proteins into flours resulted in significant improvement in flour mixing properties [measured as increased mixing time (MT) and peak resistance (PR) and as decreased breakdown in resistance], and provided a preliminary result regarding the relationships between avenin-like b proteins and functional properties of dough. When the avenin-like b proteins (containing 18 cysteine residues) were overexpressed specifically in wheat grain, whether these proteins could improve the functional properties of wheat flour still remained unclear.

In the present study, in order to confirm the effects of increasing the in vivo levels of avenin-like b proteins on the functional properties of wheat flour, the expression vector pLRPT-avel expressing specifically in the endosperm was successfully constructed and transformed into an elite wheat variety (*T. aestivum* L. cv. Zhengmai 9023) by particle bombardment. The Mixograph analysis and sodium dodecyl sulfate sedimentation (SDSS) test were performed to determine the functional properties of wheat flour using three transgenic wheat lines overexpressing the *avenin*-*like b* gene. Here we demonstrated the effects of the avenin-like b proteins overexpressed in wheat grain on the mixing properties of wheat flour.

## Materials and methods

### Plant material

Common wheat (*T. aestivum*) variety Zhengmai 9023 is a medium-strong gluten cultivar of the Yangzi River down-central area in China, with the genome constitution of AABBDD. It is a weak spring-type genotype containing four HMW-GS: Bx7, By8, Dx2 and Dy12.

### Vector construction

Genomic DNA was extracted from wheat leaves using a CTAB method (Stacey and Isaac 1994). Primers specific to the *avenin*-*like b* locus were designed since the sequences in the N- and C-terminal domains were perfectly conserved in the b-type proteins. The *avenin*-*like b* gene was amplified from the genomic DNA of wheat (*T. aestivum* cv. Zhengmai 9023) using primers containing the restriction sites *Sal*I and *Bam*HI and having the following sequences: forward primer: 5′-CGCTGTCGACATGAAGGTCTTCATCCTGGCTC-3′ (*Sal*I site underlined); reverse primer: 5′-TCGAGGATCCCTAGCACGCACCACCAGTGTA-3′ (*Bam*HI site underlined). Plasmid pLRPT was used for the construction of the transgenic expression cassette (He et al. 2005; Tosi et al. 2004). The amplified products were cloned, sequenced, digested with *Sal*I and *Bam*HI, and finally cloned into the transformation vector pLRPT, using the same restriction enzymes, resulting in its insertion between the endosperm-specific 1Dx5 promoter and the CaMV35S terminator. The recombinant vector [Electronic Supplementary Material (ESM) Fig. S1] named pLRPT-avel was co-bombarded with the plasmid pAHC25 (Christensen and Quail 1996) containing the β-glucuronidase (*uidA*) gene and the selectable marker gene *bar* that confers tolerance to phosphinothricin (PPT) under the control of maize *ubiquitin* promoter.

### Genetic transformation and plant regeneration

The transformation procedure was performed based on the bombardment method described by Sparks and Jones (2004). Immature scutella of wheat cultivar Zhengmai 9023 were used as targets for transformation by particle bombardment with the plasmids pLRPT-avel and pAHC25 at a 2:1 molar ratio. Plants were regenerated and selected under the herbicide phosphinotricin (3 mg/L) (Barro et al. 1998). Regenerated plants that survived selection were transferred to soil and grown to maturity under greenhouse conditions with air temperatures of 20/15 °C (day/night), a relative humidity of 50–70 % under ca. 350 μmol m^−2^ s^−1^ irradiance with a period of 16 h.

### PCR and Southern blotting analysis

As the *uidA* gene and CaMV35S terminator were the unique sequences in the pAHC25 vector and pLRPT-avel vector, respectively, that did not have any similarity with the genomic DNA of common wheat, PCR amplifications for the *uidA* gene (primer pair: 5′-AGTGTACGTATCACCGTTTGTGTGAAC-3′, 5′-ATCGCCGCTTTGGACATACCATCCGTA-3′) and the CaMV35S terminator (primer pair: 5′-CGCTGAAATCACCAGTCT-3′, 5′-TCCTTCCTTCCGTCCACT-3′) were used to identify transgenic plants. The amplified fragment lengths were as follows: *uidA*, 1,056 bp and CaMV35S terminator, 417 bp.

Southern blotting analysis was performed to determine the transgene copies of T_0_ plants and to indicate the independent transgenic lines. Genomic DNA was extracted from leaves of T_0_ transgenic plants by the CTAB method (Stacey and Isaac 1994) and digested with *Hin*dIII (which cuts twice within the pLRPT-avel vector). Digested genomic (10 μg) and plasmid (5 pg) DNA were separated by electrophoresis using 0.8 % (w/v) agarose gel and transferred by capillary blotting onto positively charged nylon membrane, according to the manufacturer’s instructions (Roche). The membrane was hybridized with DIG-labelled 417 bp probe generated by PCR using primers designed to the CaMV35S terminator sequence in the pLRPT-avel vector.

### SDS-PAGE and Western blotting analysis

Total proteins of wheat seeds were extracted using the method described by He et al. (1999) and separated by SDS-PAGE on 15 % polyacrylamide. Western blotting was performed using a rabbit anti-avenin-like b protein polyclonal antibody raised from the recombinant avenin-like b proteins expressed in *E. coli*. The heterologous expression of avenin-like b protein and its purification were carried out following the method reported in our previous study (Chen et al. 2008). After separation by SDS-PAGE, total proteins of wheat seeds were electro-blotted onto polyvinylidene fluoride (PVDF) membrane described by Fido et al. (1995). Primary rabbit anti-avenin-like b protein anti-serum was diluted 1:2 × 10^5^ in TBST/5 % BSA and incubated at 25 °C for 2.5 h. The membrane was washed with TBST four times and incubated with 1:4 × 10^3^ dilution of alkaline phosphatase conjugated goat anti-rabbit secondary antibody at 25 °C for 1 h, then detected according to the manufacturer’s instructions. Antibody against housekeeping protein GAPDH was used to normalize for equal amounts of proteins and to calculate the relative loading volume for each sample. The relative amounts of avenin-like b proteins in the transgenic wheat plants, compared to the non-transformed line, were measured by densitometry analysis of the Western blotting results in three biological replications using Bio-Rad Quantity One software (Bio-Rad, Hercules, CA, USA).

Single seed descent was used to obtain homozygotes for lines containing the *avenin*-*like b* transgene by PCR screening of T_3_ lines. In the following generation, SDS-PAGE analysis of avenin-like b proteins from 10 to 15 seeds of each T_4_ line was used to confirm the non-segregation of the *avenin*-*like b* gene and to determine the expression of avenin-like b proteins.

### Seed storage protein characterization

To characterize storage proteins from each line, gliadins, glutenins and other proteins were sequentially extracted from 100 mg of flour from each sample according to DuPont et al. (2005). For densitometry, the gliadin, glutenin and albumin/globulin fractions from 15 flour samples per line were separated by SDS-PAGE as described previously and quantified by densitometry method using Bio-Rad Quantity One 1-D software version 4.6.2. The densitometry method was selected because of its higher reproducibility in characterization of storage proteins than that of HPLC (Shewry et al. 2006).

### Quality tests

The protein contents and moisture contents of flour were measured by the near-infrared reflectance spectroscopy (NIRS) method using an Infratec TM1241 Grain Analyzer (Foss North America, Silver Spring, MD, USA) and samples were conditioned to 14 % moisture content and milled with a Chopin CD1 mill. The moisture contents and the protein contents of the transgenic and control lines were averaged from four replications. The water absorption was estimated by approved methods (AACC 1995) using the protein and moisture contents of flour. SDSS volumes (mL) were determined using a Brabender Quadrumat Sedimat (Brabender OHG, Duisburg, Germany) and the procedures described by ICC No. 169 (ICC Standard). Three technical replicates were carried out for each biological sample. Dough mixing properties were determined with a 10 g Mixograph (National Manufacturing Co., Lincoln, NE, USA) based on the AACC method 54-40A. Mixing was carried out in triplicate. The mixing parameters determined were MT (min), the maximum height of the midline trace (PR [arbitrary units, AU]), bandwidth at peak resistance (BWPR [AU]), percentage decrease in dough resistance 3 min after the peak (resistance to breakdown, RBD [%]), the maximum bandwidth during the mixing (MBW [AU]), the bandwidth of the midline after MT (MRW [AU]) and the midline integral at 8 min (MTxI [AU]).

### Extraction of monomeric proteins, soluble and insoluble polymeric glutenin

Alcohol-soluble gluten proteins were extracted with 50 % (v/v) propan-1-ol in a 1:10 (mg:μL) ratio using the method described by Tosi et al. (2005). Insoluble glutenin proteins were extracted from the residue using a buffer containing 0.07 M Tris–HCl pH 6.8, 2 % (w/v) SDS, 10 % (w/v) glycerol, 0.002 % (w/v) bromophenol blue and 1 % (w/v) dithiothreitol in the same weight:volume ratio. Ten μL aliquots of the soluble and insoluble extracts were analyzed by SDS-PAGE using the standard method with 10 % separating gels, and Western blotting analysis.

### SE-HPLC

Size exclusion-high performance liquid chromatography (SE-HPLC) was carried out using an Agilent 1100 Series chromatography station with a UV detector set at 214 nm. The total proteins were extracted following the method described by Tosi et al. (2005). The supernatants were filtered through a 0.45 μm membrane and 20 μL were injected into a ZORBAX GF-250 size-exclusion column, with temperature controlled at 40 °C. A 1:1 mixture of 0.05 % trifluoroacetic acid (TFA) in acetonitrile and 0.05 % TFA in deionized water was used as eluting solvent at a flow rate of 0.5 mL/min. Samples were extracted in three replicates and two separations of each extraction made.

The soluble proteins and the insoluble proteins in flour were extracted according to the method described by Tosi et al. (2005). In both cases the supernatants were filtered through 0.45 μm PVDF filters and 20 μL were injected into a ZORBAX GF-250 size-exclusion column, under the same condition described for total proteins. Three replicate separations were performed on each flour sample. The proportion of total polymeric proteins (%UPP) was determined according to Gupta et al. (1993).

### Statistical analysis

Statistical analysis was performed using SPSS for Windows 15.0 statistical software (SPSS Inc.,). Significance of means within each data set was determined by Fisher’s least significant difference (LSD) test with *p* = 0.05.

## Results

### Production of transgenic wheat plants

The *avenin*-*like b* gene sequence in this research was 855 bp long with the GenBank accession number HM027637 and encoded a protein with 284 amino acid residues containing 18 cysteine residues, being grouped into avenin-like b (typ-b3) proteins (Kan et al. 2006). The phylogenetic relationships of avenin-like b proteins have been analyzed in our previous study (Chen et al. 2008). The first 18 amino acid residues corresponded to the signal peptide and the mature protein contained 266 amino acid residues having an average relative molecular mass of 30 kDa (De Caro et al. 2010). Plasmid pAHC25 conferring bialaphos resistance and plasmid pLRPT-avel encoding avenin-like b protein were used for transformation of the wheat variety Zhengmai 9023.

**Fig. 1:**
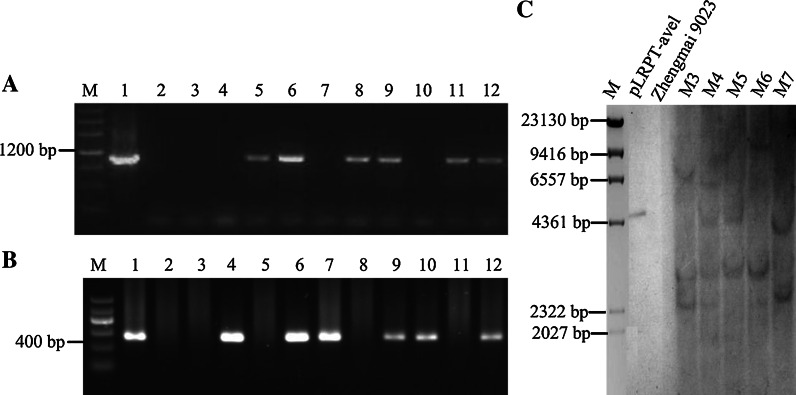
PCR (**a**, **b**) and Southern blotting analysis (**c**) of the transgenic wheat plants. *Left* PCR amplification results of *uidA* gene (**a**) and CaMV35S terminator sequence (B). *Lane M* DNA marker III (**a**) or marker II (**b**); *lane 1* plasmid pAHC25 (**a**) or pLRPT-avel (**b**) for positive control; *lane 2* water for negative control; *lane 3* DNA of cv. Zhengmai 9023 for negative control; *lanes 4*–*12* DNA of regenerated wheat plants. The samples in *lanes 4*–*12* of **b** correspond to the samples in *lanes 4*–*12* of **a**, respectively. *Right* Southern blotting analysis (**c**) of *Hin*dIII-cut genomic DNA from transgenic wheat lines (*M3*–*M7*) and from non-transformed control line (cv. Zhengmai 9023), hybridized with a probe prepared by random priming of the CaMV35S terminator sequence. *Lane M* λDNA/*Hin*dIII marker. Positive control of pLRPT-avel digested with *Hin*dIII. The PCR results of transgenic wheat lines (*M3*–*M7*) in **c** are shown in *lanes 4, 6, 7, 9* and *12* of **b**, respectively

The positive transgenic T_0_ plants were confirmed by PCR to amplify the *uidA* gene and CaMV35S terminator sequence (Fig. 1a, b). A total of 30 positive transgenic T_0_ plants were obtained from about 3,000 bombarded immature scutella. The overall transformation efficiency was 1 %. The offspring of some transgenic T_0_ plants were further confirmed on the basis of Southern and Western blotting analyses.

Southern blotting analysis using *Hin*dIII to digest the recombinant plasmid pLRPT-avel and the genomic DNA of transgenic wheat lines and non-transformed line hybridized with a probe corresponding to the CaMV35S terminator sequence showed that the selected T_0_ transgenic wheat lines contained multiple insertion sites (Fig. 1c), resulting in two or more bands on the blot. The banding patterns, however, were different, confirming that the plants were derived from independent transformation events and could be therefore considered as independent lines (Fig. 1c). The corresponding relationships of the samples in Fig. 1a–c are indicated in the Fig. 1 caption.

Recombinant plasmid pLRPT-avel was digested with *Hin*dIII (cuts twice within the plasmid), known to excise a ~4.6 kbp fragment including the transgene and the probe sequence of CaMV35S terminator (ESM Fig. S1). As shown in Fig. 1c, hybridizing fragments of the expected size, which were presumed to correspond to full-length versions of the expression vector digested with *Hin*dIII (~4.6 kbp), were detected in genomic DNA from the transgenic M4 and M5 lines. Moreover, hybridizing bands of the larger or smaller sizes than the expected band (~4.6 kbp), probably derived from transgene rearrangement and/or modification, were also detected in the transgenic M4 and M5 lines.

However, hybridizing bands of larger or smaller sizes than the expected size (~4.6 kbp) were also detected in the transgenic M3, M6 and M7 lines. This was most likely because the expression plasmid insertions had undergone deletions or rearrangements resulting in the loss of either *Hin*dIII restriction sites or the sequence of the expression plasmid in the process of genetic transformation. The presence of the larger or smaller than expected sizes in Southern blotting was similar to the results of previous studies (Zhang et al. 2003; Tosi et al. 2004; He et al. 2005).

Selection of homozygous progeny with stable expression of avenin-like b proteins was carried out in four generations (T_0_–T_3_) by SDS-PAGE and Western blotting analysis. In addition, the presence of *uidA* gene and CaMV35S terminator were determined by PCR in four generations. Three homozygous transgenic wheat lines (designated M3, M6 and M7) in the T_3_ generation overexpressing avenin-like b proteins were obtained, while the other transgenic lines tested were still hemizygous. Mature T_4_ seeds were harvested separately from each plant and analyzed by SDS-PAGE and Western blotting to verify the stability of transgene expression and homozygosity. Three homozygous lines overexpressing *avenin*-*like b* gene (named M3, M6 and M7), together with one non-transgenic line (designated N-4) derived from T_1_ hemizygous plants, and one non-transformed line (cv. Zhengmai 9023), were used for further analysis.

**Fig. 2:**
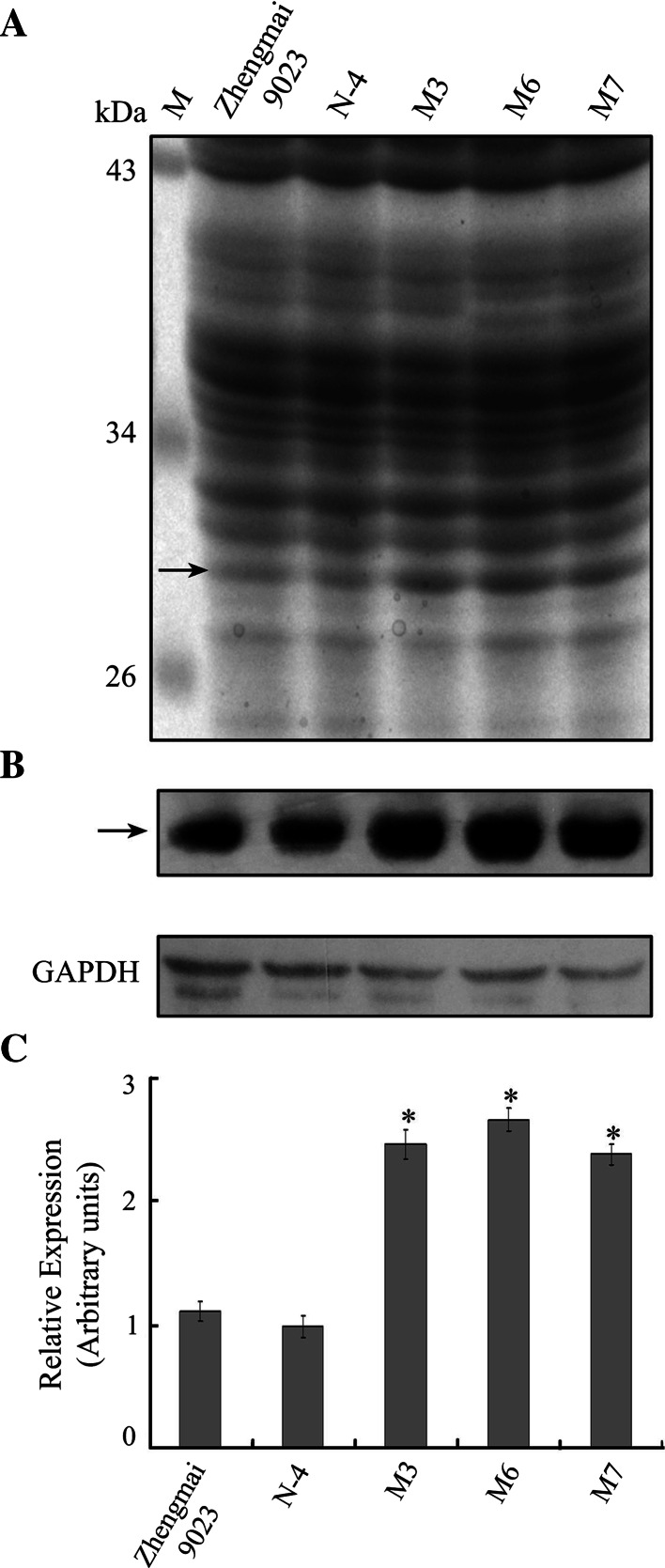
SDS-PAGE (**a**) and Western blotting analysis (**b**) of avenin-like b proteins in transgenic lines (*M3*, *M6* and *M7*) and control lines (cv. Zhengmai 9023 and N-4). **a** *Lane M* protein marker. *Arrow* indicates the position of the transgenic avenin-like b proteins. **b** Western blotting results of avenin-like b proteins in transgenic and control lines. Housekeeping protein GAPDH was used as control to normalize for equal amounts of proteins and to calculate the relative loading volume for each sample. **c** Relative amounts of the avenin-like b proteins in the transgenic plants were densitometrically quantified with respect to the non-transformed control line (cv. Zhengmai 9023)

Total seed storage protein of the transgenic (M3, M6 and M7) lines and control (cv. Zhengmai 9023 and N-4) lines were separated by SDS-PAGE for identification of the transgenic subunits by staining with Coomassie Brilliant Blue R250. The amounts of avenin-like b proteins in the three transgenic wheat lines were increased compared with the non-transformed control line (Fig. 2a, arrow). Due to the high sensitivity of the Western blotting analysis, it has been possible to assess more accurately the avenin-like b proteins expressed in wheat grain. Western blotting analysis was carried out using polyclonal antibodies to confirm whether the amounts of avenin-like b proteins in the transgenic wheat lines were increased. A clear reactive band of the expected molecular mass (about 30 kDa) was observed in all seed protein extracts (Fig. 2b). The relative amounts of avenin-like b proteins in the transgenic wheat, compared to the non-transformed line, were measured by using GAPDH as control to normalize for equal amounts of proteins and to calculate the relative loading volume for each sample. Western blotting analysis showed that the relative amounts of avenin-like b proteins in three transgenic M3, M6 and M7 lines were increased 1.6-, 1.8- and 1.4-fold, respectively, when compared with the non-transformed line, as calculated by densitometry (Fig. 2c). However, no significant difference in the amount of avenin-like b proteins was observed between the non-transgenic line (N-4) and non-transformed control line (cv. Zhengmai 9023) (Fig. 2). These results demonstrated that the amount of avenin-like b proteins was not changed in line N-4 by possible somaclonal variations in the process of genetic transformation.

### Characterization of the storage proteins in the transgenic and control lines

The flour protein contents varied from 13.57 % in the non-transformed lines and 13.79 % in the non-transgenic lines to 14.33, 14.61 and 14.47 % in the M3, M6 and M7 lines, respectively. Although flour protein contents for the transgenic lines were higher than those of the control lines, this increase in protein contents was not significant as determined by analysis of variance (ANOVA) analysis. Further, SDS-PAGE results of total storage protein did not show a difference in their expression patterns among transgenic and control lines (ESM Fig. S2A).

To compare the protein compositions of flour samples from these lines, we separated and quantified glutenins and gliadins according to DuPont et al. (2005). ANOVA analysis results showed that differences in the amounts of glutenins and gliadins were not significant between these lines (ESM Fig. S2B). As shown in ESM Fig. S2, overexpression of avenin-like b proteins did not influence the amounts and proportions of storage proteins. SDS-PAGE analysis of total proteins showed no marked difference in the endogenous HMW-GS (1Dx2, 1Bx7, 1By8 and 1Dy12) subunits of transgenic and control lines. The proportions of HMW-GS in the glutenin varied from 17.32 to 17.43 % for line M6 and the non-transformed control line, while those of LMW-GS ranged from 82.68 to 82.81 % for line M6 and the non-transformed control line. Furthermore, the ratios of HMW/LMW were similar between transgenic and control lines. In general, the expression of the avenin-like b proteins in the transgenic lines did not alter the amounts and proportions of glutenins (including both HMW- and LMW-GS) and gliadins in the endosperms of wheat.

### Mixing properties analysis

**Fig. 3:**
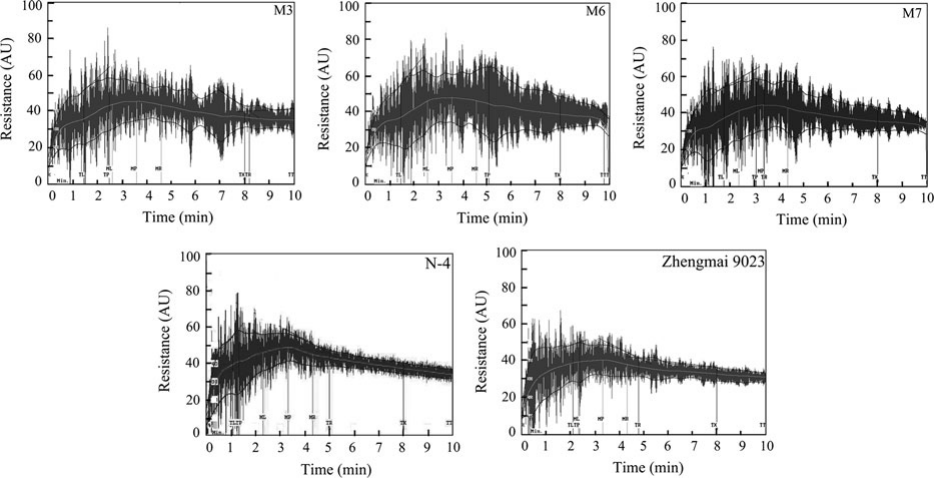
Mixograph curves of the dough prepared from three transgenic wheat lines (*M3*, *M6* and *M7*) and control lines (N-4 and cv. Zhengmai 9023). *TL* time before peak of trace envelope, *TP* time to peak of trace envelope, *TR* time after peak of trace envelope, *ML* time before peak of the midline of the trace, *MP* time to peak of the midline of the trace, *MR* time after peak of the midline of the trace, *TX* time at 8 min of mixing, *TTT* time at 10 min of mixing

The SDSS test showed no differences between the two control lines (N-4 and cv. Zhengmai 9023). The average SDSS volume was significantly higher in the three transgenic lines (M3, M6 and M7) than in the control lines (Table 1). The mixograms and mixing parameters of dough from the three transgenic wheat lines and control lines, measured using a 10 g Mixograph, with three replications, are shown in Fig. 3 and Table 1, respectively. No significant differences were found in the mixing parameters between the non-transgenic line (N-4) and non-transformed line (cv. Zhengmai 9023), suggesting that particle bombardment and tissue culture of wheat did not affect dough mixing properties (Table 1).

**Table 1:** Comparisons of flour quality-related parameters of the transgenic and control wheat lines

Parameters	Lines					LSD 0.05
	Zhengmai 9023	N-4	M3	M6	M7	
*Mixograph*						
Mixing time (min)	3.42 ± 0.09^a^a	3.35 ± 0.05a	3.46 ± 0.04a	3.56 ± 0.04a	3.45 ± 0.03a	NS
Peak resistance (AU)	40.28 ± 0.14a	42.35 ± 0.37a	45.67 ± 0.78b	46.16 ± 0.67b	45.38 ± 0.58b	2.46
Resistance breakdown (%)	16.42 ± 0.76a	17.34 ± 0.52a	14.44 ± 0.67b	13.16 ± 0.44b	13.57 ± 0.36b	1.68
Bandwidth at peak resistance (AU)	17.2 ± 0.43a	15.54 ± 0.69a	26.44 ± 0.65b	24.92 ± 0.48b	27.06 ± 0.51b	2.17
Bandwidth of the midline after mixing time (AU)	18.62 ± 1.81a	13.06 ± 0.78a	28.98 ± 0.66b	25.77 ± 0.74b	28.33 ± 0.96b	5.96
Maximum bandwidth during the mixing (AU)	21.17 ± 0.21a	27.98 ± 1.35ac	31.73 ± 1.26bc	35.91 ± 3.5b	33.64 ± 2.17b	6.04
Midline integral at 8 min (AU)	282.48 ± 3.57a	291.58 ± 0.78a	314.23 ± 4.66b	322.36 ± 3.74b	319.35 ± 2.96b	9.62
*SDSS test*						
Sedimentation (mL) at 14 %	41.5 ± 0.1a	41.7 ± 0.3a	52.77 ± 0.1b	54.12 ± 0.3b	53.72 ± 0.1b	1.62
*SE*-*HPLC*						
%F1	36.64 ± 0.3a	37.69 ± 0.43a	46.31 ± 1.63b	47.29 ± 0.63b	46.82 ± 0.78b	2.17
%F1/%F2	2.11 ± 0.17a	2.15 ± 0.13a	2.38 ± 0.11b	2.6 ± 0.15b	2.41 ± 0.19b	0.25
(%F3 + %F4)/%F1	1.19 ± 0.03a	1.07 ± 0.02a	0.74 ± 0.04b	0.73 ± 0.03b	0.79 ± 0.05b	0.28
%UPP^b^	35.57 ± 0.41a	36.56 ± 1.02a	43.53 ± 1.32b	44.1 ± 1.54b	43.87 ± 1.28b	3.52

Values within the same parameter followed by the same letter are not significantly different at 0.05 probability level

*NS* not significant, *AU* arbitrary units, *LSD 0.05* least significant difference at *p* = 0.05

^a^Mean ± SD among three replications

^b^%UPP (polymeric insoluble fraction/total polymeric protein) of the transgenic and control line flour

As shown in Table 1 and Fig. 3, the MBW were significantly higher in the three transgenic wheat lines M3 (31.73), M6 (35.91) and M7 (33.64) than in the non-transformed lines (21.17). The MRW varied from 18.62 in the non-transformed lines to 28.98, 25.77 and 28.33 in the transgenic lines M3, M6 and M7, respectively. All the dough from the transgenic wheat lines had higher PR and broader BWPR than the non-transformed lines (Fig. 3). The PRs of transgenic wheat M3, M6 and M7 lines were increased to 45.67, 46.16 and 45.38, respectively when compared to that of the non-transformed line (40.28). Moreover, the dough from transgenic lines had thicker BWPR than the dough from the non-transformed lines (Fig. 3), suggesting that the resistance to extension of the transgenic wheat dough was improved (Tosi et al. 2005). Based on the RBD (Table 1), the mixing tolerance of the transgenic wheat dough was improved. The RBD of transgenic wheat lines M3, M6 and M7 were decreased to 14.44, 13.16 and 13.57, respectively, compared to 16.42 in the non-transformed wheat lines. Furthermore, the increased MTxI for transgenic lines indicated the enhancement of dough strength in comparison with those for control lines. In addition, slight positive changes were observed in MT. The MT of transgenic lines M3, M6 and M7 was 0.04, 0.14 and 0.03 min higher, respectively, when compared with the non-transformed lines, but these differences were not statistically significant. It could be concluded that the overexpression of avenin-like b proteins generally led to improved dough elasticity, mixing tolerance and dough resistance to extension.

### Analysis of glutenin polymers in the transgenic and control lines

Gluten proteins are classically divided into two groups, the gliadins and glutenins, based on their extractability (albumins, globulins and gliadins) or unextractability (glutenin) in aqueous alcohols (Shewry and Tatham 1997). However, small amounts of polymers related to the glutenins are also present in the gliadin fraction. These appear to differ from the alcohol-unextractable glutenins in having lower molecular mass and higher amounts of LMW subunits, and have been called ‘aggregated gliadins’ (Shewry et al. 1983), ‘high molecular-weight gliadin’ or ‘ethanol-soluble glutenin’ (Bietz and Wall 1980). In this study, monomeric, soluble and insoluble polymeric glutenin proteins of wheat flour were extracted following the method described by Tosi et al. (2005). Proteins were separated into fractions soluble (soluble gluten protein, 50PS) and insoluble (insoluble gluten protein, 50PI) in 50 % (v/v) propan-1-ol. The 50PI fraction was essentially free of monomeric proteins and comprised mainly glutenin, while the 50PS fraction was a mixture of monomeric proteins and soluble polymeric glutenin (Bean et al. 1998; Fu and Sapirstein 1998; Sapirstein and Fu 1996). We therefore initially determined the effects of the avenin-like b proteins on the proportions of alcohol-soluble and alcohol-insoluble proteins and on the distribution of transgenic subunits in these fractions.

**Fig. 4:**
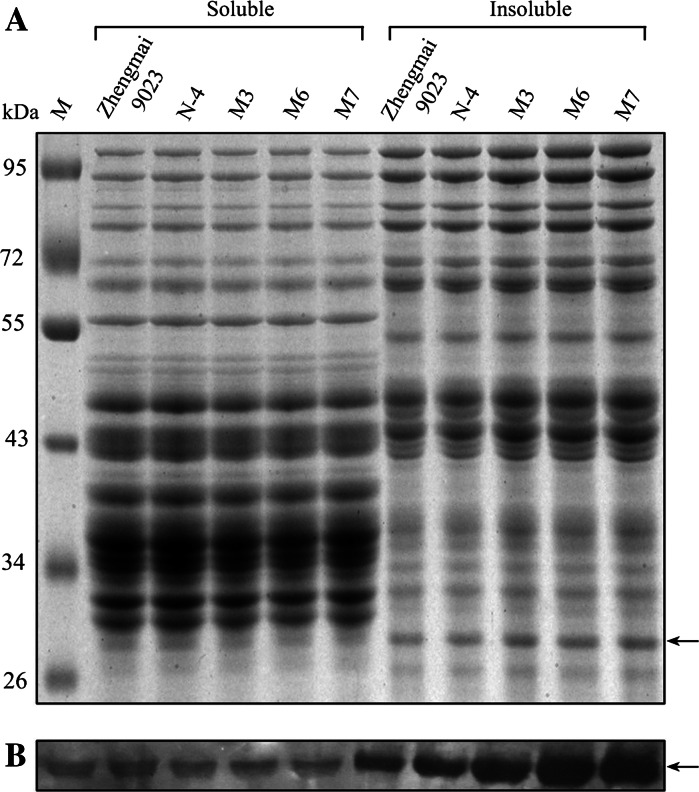
SDS-PAGE (**a**) and Western blotting analysis (**b**) of soluble and insoluble gluten protein fractions extracted from flour of non-transformed line (cv. Zhengmai 9023), non-transgenic line (N-4) and transgenic lines (*M3*, *M6* and *M7*). *Lane M* protein marker. *Arrow* indicates the position of the transgenic avenin-like b proteins

The SDS-PAGE patterns under reducing conditions of these fractions from the five flour samples are shown in Fig. 4. Gel scanning was used to determine the relative proportions of these two fractions (by determining the total absorbance of the tracks). The ratios of soluble to insoluble proteins were significantly lower in the three transgenic lines than in the control lines. The ratios of soluble to insoluble proteins varied from 1.42 in the non-transformed lines and 1.38 in the non-transgenic lines to 1.19, 1.12 and 1.08 in the M3, M6 and M7 lines, respectively. In our study, no significant difference in the amount of avenin-like b proteins was observed in the 50PS and 50PI fractions between the non-transgenic line (N-4) and non-transformed line (cv. Zhengmai 9023) (Fig. 4). This result met expectation, as line N-4 showed expression amounts of avenin-like b proteins in the wheat grain almost identical to the non-transformed line (Fig. 2; ESM Fig. S2).

Figure 4a showed that no significant difference in the amount of avenin-like b proteins in the 50PS fraction was observed between the control lines and transgenic lines (M3, M6 and M7), while the amounts of avenin-like b proteins in the 50PI were increased in the transgenic lines compared with those of the control lines (Fig. 4, arrow). Especially in transgenic lines M6 and M7, avenin-like b proteins were much more abundant in the polymers than in the transgenic line M3. These results indicated that the overexpression of avenin-like b proteins in transgenic wheat lines was presumably incorporated into the polymers.

SE-HPLC can be used to fractionate gluten proteins based on their molecular masses without the reduction of the inter-chain disulphide bonds that stabilized the glutenin polymers. The chromatographic patterns were divided into four fractions corresponding to large polymeric proteins (F1), smaller polymeric proteins (F2), large monomeric proteins (F3) and smaller monomeric proteins (F4), respectively (Tosi et al. 2005). Each fraction was quantified by measurement of the peak area. The relative amounts of the individual fractions were quantified as the percentages of each peak relative to the total area and the four separated fraction amounts were expressed as %F1, %F2, %F3 and %F4, respectively. The relative amounts of the peaks do relate to dough strength, with %F1/%F2 and (%F3 + %F4)/%F1 showing particularly strong correlations (Morel et al. 2000). The SE-HPLC results for the transgenic and control lines are summarized in Table 1.

As judged from the SE-HPLC analysis, the %F1 was drastically increased in the transgenic wheat lines compared to the control lines. The %F1 amounts in the M3, M6 and M7 lines were increased 0.26, 0.29 and 0.28 times respectively. All transgenic lines had higher values for %F1/%F2 while the values for (%F3 + %F4)/%F1 were decreased in the three transgenic lines compared with the control lines (Table 1), indicating that all the transgenic lines had higher proportions of polymeric proteins. This observation was also supported by determination of the proportion of unextractable polymeric protein (%UPP), which represented an alternative way of measuring polymer size distribution. The %UPP in the three transgenic lines M3 (43.53 %), M6 (44.1 %) and M7 (43.87 %) were markedly higher than in the non-transformed wheat lines (35.57 %) (Table 1). Based on the above results, the increased amounts of avenin-like b protein resulted in a significant effect on the proportion of polymeric proteins.

## Discussion

The objective of this study was to evaluate the effect of avenin-like b proteins on the functional properties of wheat flour. We have clearly demonstrated that all the transgenic lines that tested PCR-positive for the *uidA* gene and CaMV35S terminator were also positive for transgene expression, unequivocally determined by Western blotting with the anti-avenin-like b protein polyclonal antibody. After selection for four generations, three transgenic wheat lines overexpressing the *avenin*-*like b* gene (designated M3, M6, and M7), one non-transgenic line (designated N-4) and one non-transformed control line (cv. Zhengmai 9023) were used to investigate the relationships between avenin-like b proteins and the mixing properties of wheat flour.

### Overexpression of avenin-like b proteins has positive effects on dough mixing properties

In this work, both total proteins and dough mixing properties were characterized (Fig. 3; ESM Fig. S2). Due to the dominant role of glutenins in dough functionality, we first determined whether any difference in the glutenin profiles existed between transgenic lines and control lines. As shown in ESM Fig. S2, three transgenic lines, the non-transgenic line and the non-transformed line showed almost identical SDS-PAGE patterns of storage proteins, indicating that overexpression of avenin-like b proteins did not alter any aspects of the glutenin profiles.

The potential bread-making quality of the flours was assessed with two small-scale tests: the SDSS test and Mixograph analysis (Carter et al. 1999; Lorenzo and Kronstad 1987; Martinant et al. 1998). SDSS volume is a predictor of baking quality that is often used by breeders to screen small flour samples (Dick and Quick 1983). The results reported here showed significant increases in the average of SDSS volumes of the transgenic lines. According to the SDSS values, the transgenic lines had better quality than their control lines. High SDSS volumes have been associated with stronger gluten and superior bread-making quality (Ayoub et al. 1993; Lorenzo and Kronstad 1987). Mixing properties of transgenic lines analyzed using the 10 g Mixograph provided information on dough gluten strength, closely correlated with baking quality. The relationships between Mixograph parameters and dough viscoelasticity have been explained in detail (Martinant et al. 1998) and associations of these parameters with other wheat quality traits have also been discussed previously (Bordes et al. 2008). In generally, MPW, MRW, BWPR and MBW are positively correlated with dough resistance to extension (Tosi et al. 2005), while RBD is negatively correlated with the mixing tolerance (Tosi et al. 2005; Piston et al. 2011). MT, MPV and MTxI are positively correlated with dough strength (León et al. 2009). Weak dough has higher RBD, shorter MT, lower PR and smaller MTxI when compared to strong dough (León et al. 2009; Piston et al. 2011; Li et al. 2012). In our study, no significant difference in mixing parameters was observed between the non-transgenic line (N-4) and non-transformed control line (cv. Zhengmai 9023) (Fig. 3; Table 1). This result met expectation, as line N-4 showed almost identical patterns of storage proteins with the non-transformed control line (ESM Fig. S2). These results demonstrated that no variations in storage proteins were caused in line N-4 by possible somaclonal variations in the process of genetic transformation.

We further compared the mixing parameters of transgenic lines (M3, M6 and M7), the non-transgenic line (N-4) and the non-transformed control line (cv. Zhengmai 9023). In transgenic lines, the significant increase of PR demonstrated that dough elasticity was increased by overexpression of the *avenin*-*like b* gene. Significantly lower RBD values for lines M3, M6 and M7 revealed that the mixing tolerance was also improved. In transgenic lines, all mixing parameters relating to the curve widths showed significant increases compared to the control lines, indicating that dough resistance to extension was improved. Furthermore, significant increases in MT, PR and MTxI were detected in the transgenic lines, suggesting that the overexpression of avenin-like b proteins caused an increase in dough strength. It could be concluded that overexpression of avenin-like b proteins generally led to improved dough elasticity and mixing tolerance and increased dough resistance to extension.

These results showed a similar trend to the results of our previous report, that incorporation of avenin-like b proteins into flour resulted in statistically significant increases in PR and mixing tolerance by in vitro assay (Chen et al. 2010), indicating that avenin-like b proteins had advantageous effects on mixing properties of wheat flour. In this study, the difference in MT between the transgenic wheat lines and control lines was not statistically significant, while Chen et al. (2010) reported that statistically significant increases were observed in MT by incorporation of avenin-like b proteins into flour. This difference might come from the different protein sequence, different wheat cultivars, the unknown (undetermined) amount of avenin-like b protein in transgenic wheat lines and different mixing equipment used for these studies.

### Incorporation of avenin-like b proteins into the glutenin polymers

The avenin-like b proteins of this research contained 18 cysteine residues, speculated to form inter-chain disulphide bonds allowing incorporation into high-molecular-mass polymers (Kan et al. 2006). In order to confirm this possibility, monomeric, soluble and insoluble polymeric glutenin proteins of wheat flour were extracted using the method described by Tosi et al. (2005). Both the soluble and insoluble extracts were analyzed by SDS-PAGE and Western blotting analysis. These results showed that avenin-like b proteins existed in both 50PS (monomericn and soluble polymeric glutenin) and 50PI (insoluble polymer proteins). Moreover, the amount of avenin-like b proteins in the insoluble polymer proteins was increased in the transgenic lines (M3, M6 and M7) compared to the control lines, while no significant differences were found in the soluble fractions between the three transgenic lines and control lines (Fig. 4). Based on these results, it was reasonable to conclude that transgenic avenin-like b proteins were indeed incorporated into the glutenin polymers.

This observation was also supported by the results of SE-HPLC analysis which was used to analyze the polymer size distribution between transgenic and control lines. Based on the SE-HPLC analysis (Table 1), the overexpression of avenin-like b proteins in the transgenic lines could result in significant effects on the proportion of the polymeric gluten protein fractions, indicating that the avenin-like b proteins affected the degree of cross-linking. Furthermore, the %UPP in the three transgenic lines (M3, M6 and M7) were markedly higher than in the control lines (Table 1). On the basis of the above analysis, the increased amounts of avenin-like b proteins could result in significant effects on the proportion of the polymeric gluten protein fractions. These results demonstrate that the improvement in flour properties in the transgenic lines was associated with an increased proportion of polymer proteins.

Our results showed that the overexpression of avenin-like b proteins in transgenic wheat lines improved flour mixing properties and demonstrated that the overexpression of avenin-like b proteins in transgenic plants led to an increase in the proportion of large polymeric proteins, which should help to understand the influence and mechanism of avenin-like b proteins on the functional properties of wheat flour. These results also suggested that avenin-like b proteins play an important role in determining the mixing properties of wheat flour and that the *avenin*-*like b* gene could be a candidate gene for improving functional properties of wheat flour. It would be of interest to clarify whether different types of avenin-like b proteins with different numbers and/or positions of cysteine residues could improve the mixing properties of dough in the same way. Therefore, more work is required for a better understanding of their role in the functional properties of wheat flour in the future.

## Electronic supplementary material

Below is the link to the electronic supplementary material.

Supplementary Fig. S1 Map of the plasmid pLRPT-avel with the positions of relevant restriction sites. The *avenin*-*like b gene* was inserted between the endosperm-specific 1Dx5 promoter and the CaMV35S terminator (TIFF 552 kb)

Characterization of storage proteins in transgenic (M3, M6 and M7) lines and control (cv. Zhengmai 9023 and N-4) lines. A. SDS-PAGE of seed protein extracts from transgenic lines, non-transgenic line and non-transformed control line. Arrow indicates the position of the transgenic Avenin-like b proteins. B. Characterization of storage proteins from the transgenic and control lines. HMW % glutenin and LMW % glutenin means quantities of HMW-GS and LMW-GS, respectively, expressed relative to total quantity of the glutenins (and the same for 1Dx2 %, 1Bx7 %, 1By8 %, and 1Dy12 %). HMW/LMW: ratio of the high and low molecular weight glutenin subunits. Glutenin %: quantity of the glutenins expressed relative to total proteins extracted by the sequential extraction methods (and the same for Gliadin %). Glu/Glia: ratio of the glutenins and gliadins. Data are given as mean ± SEM. Values within the same characteristics of storage proteins are not significantly different (P =0.05) (TIFF 1913 kb)

## Abbreviations

BWPR: Bandwidth at peak resistance
MBW: Maximum bandwidth during the mixing
MRW: Bandwidth of midline after mixing time
MT: Mixing time
MTxI: Midline integral at 8 min
NFDM: Non-fat dry milk
PR: Peak resistance
PVDF: Polyvinylidene fluoride
RBD: Resistance to breakdown
SDSS: Sodium dodecyl sulfate sedimentation
SE-HPLC: Size exclusion-high performance liquid chromatography
