# Supplementary figures and images for: Transformation of common wheat (Triticum aestivum L.) with avenin-like b gene improves flour mixing properties
Source: Mol Breed. 2013 Aug 2;32(4):853–65. doi: 10.1007/s11032-013-9913-1 (PMC3830129; doi:10.1007/s11032-013-9913-1)

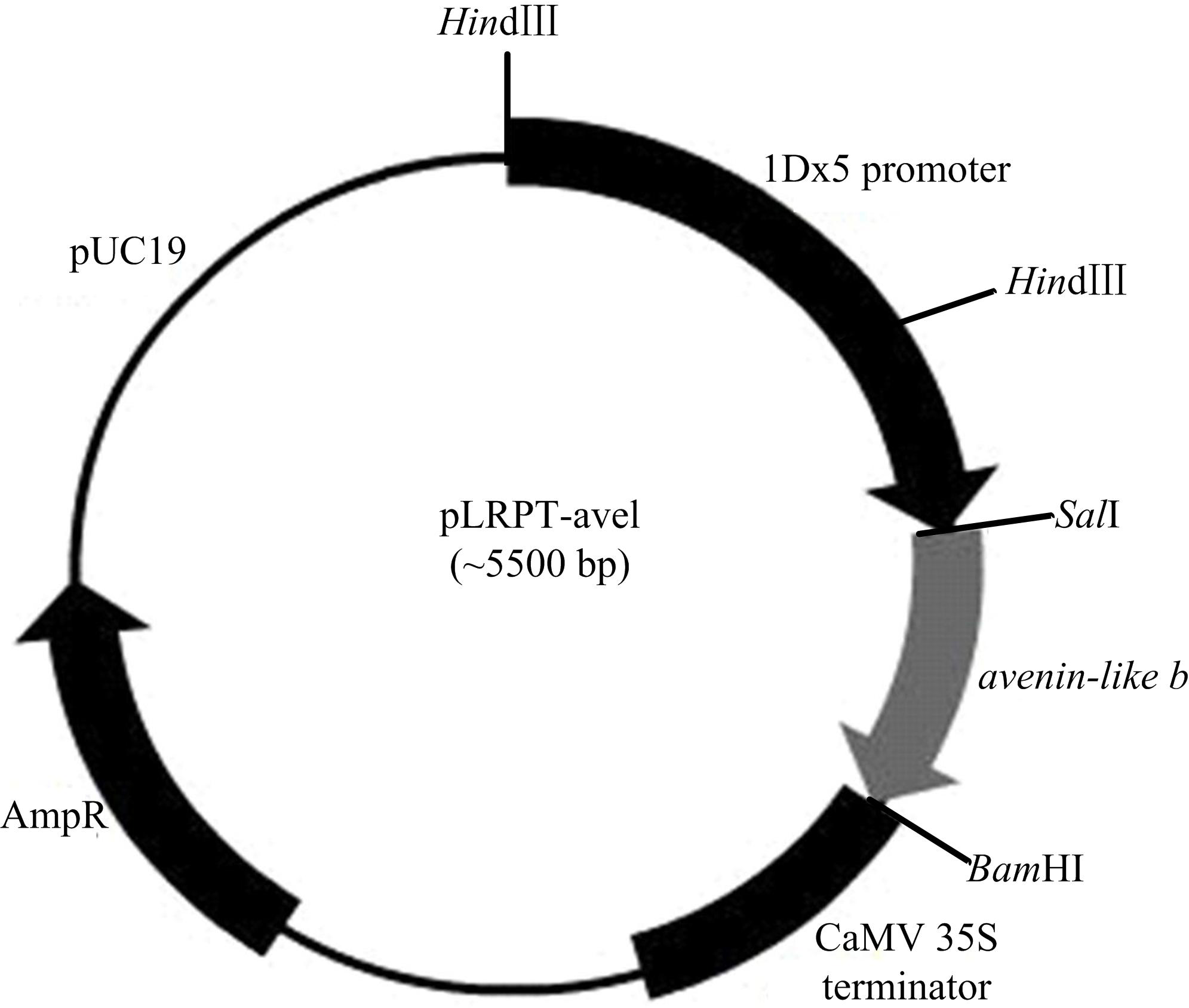

Supplement: Supplementary file 1 — Supplementary Fig. S1 Map of the plasmid pLRPT-avel with the positions of relevant restriction sites. The avenin-like b gene was inserted between the endosperm-specific 1Dx5 promoter and the CaMV35S terminator (TIFF 552 kb) [file 11032_2013_9913_MOESM1_ESM.tif]

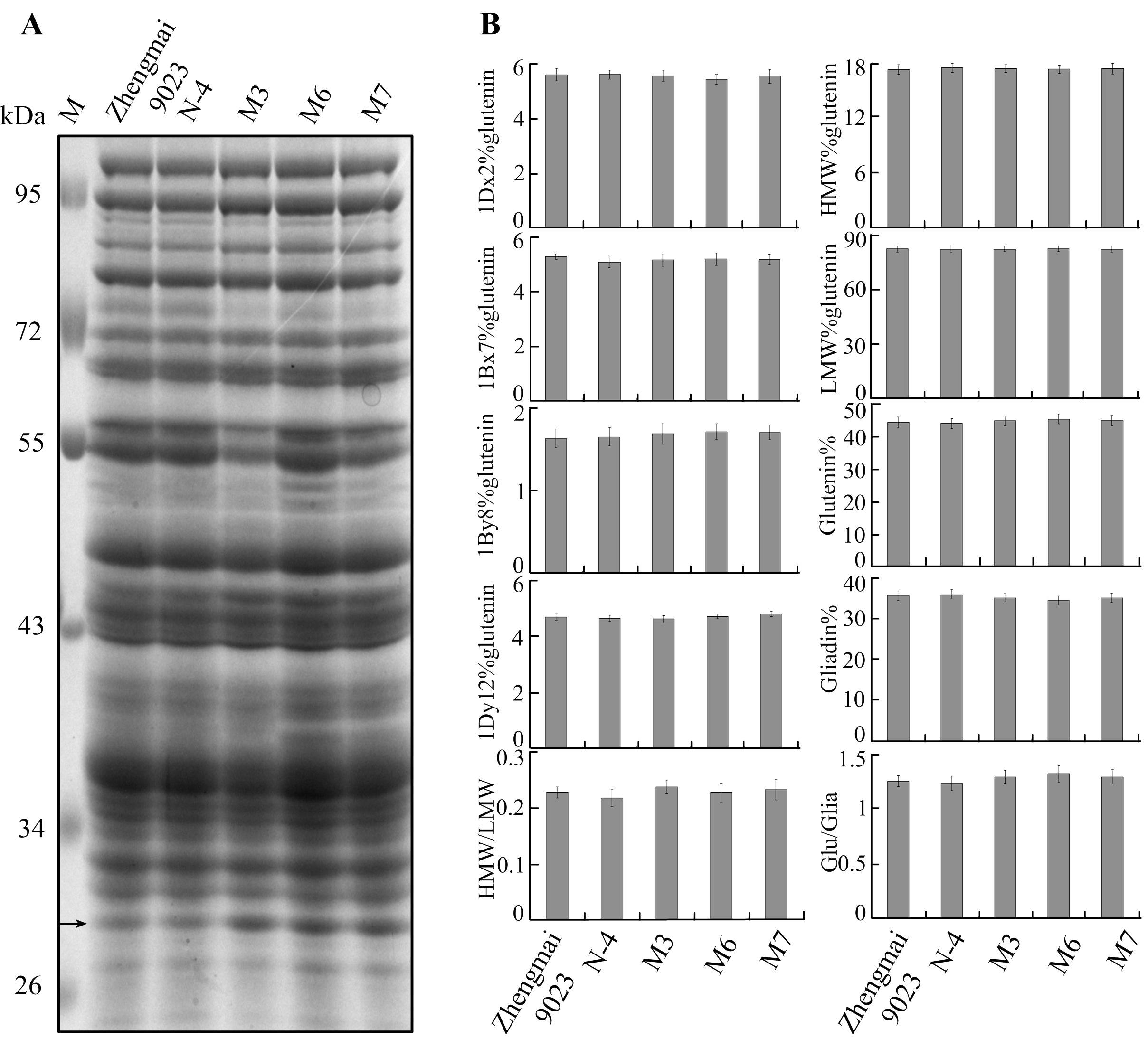

Supplement: Supplementary file 2 — Characterization of storage proteins in transgenic (M3, M6 and M7) lines and control (cv. Zhengmai 9023 and N-4) lines. A. SDS-PAGE of seed protein extracts from transgenic lines, non-transgenic line and non-transformed control line. Arrow indicates the position of the transgenic Avenin-like b proteins. B. Characterization of storage proteins from the transgenic and control lines. HMW % glutenin and LMW % glutenin means quantities of HMW-GS and LMW-GS, respectively, expressed relative to total quantity of the glutenins (and the same for 1Dx2 %, 1Bx7 %, 1By8 %, and 1Dy12 %). HMW/LMW: ratio of the high and low molecular weight glutenin subunits. Glutenin %: quantity of the glutenins expressed relative to total proteins extracted by the sequential extraction methods (and the same for Gliadin %). Glu/Glia: ratio of the glutenins and gliadins. Data are given as mean ± SEM. Values within the same characteristics of storage proteins are not significantly different (P =0.05) (TIFF 1913 kb) [file 11032_2013_9913_MOESM2_ESM.tif]
